# Recurrent Pheochromocytoma in an Elderly Patient

**DOI:** 10.3390/medicina56060316

**Published:** 2020-06-26

**Authors:** Ammu Thampi Susheela, Howide Eldib, Deepthi Vinnakota, Andrea Bial, Salman Ali, Hannah Koh, Brian Lavery, Martin Gorbien

**Affiliations:** Loyola Medical Center/Edward Hines, Jr. VA Hospital, Maywood, IL 60141, USA; Howide.Eldib@va.gov (H.E.); rdvinnakota@gmail.com (D.V.); Andrea.Bial@va.gov (A.B.); Salman.Ali@va.gov (S.A.); Hannah.Koh@va.gov (H.K.); Brian.Lavery@va.gov (B.L.); Martin.Gorbien@va.gov (M.G.)

**Keywords:** Pheochromocytoma, neuroendocrine tumor

## Abstract

Pheochromocytomas are rare neuroendocrine tumors that can affect people of all ages and are commonly diagnosed in the 4th and 5th decades of life. Familial pheochromocytomas are diagnosed mostly between the 2nd and 3rd decades of life. They can be benign or metastatic and often present as isolated tumors or along with other neuroendocrine syndromes. We present a case of an elderly man who underwent laparoscopic adrenalectomy for pheochromocytoma at the age of 60 years but developed recurrence of metastatic pheochromocytoma after ten years. We also conducted a literature review to understand the epidemiology and presentation of the tumor and to emphasize that there should be a low threshold of suspicion for timely diagnosis and management of recurrent pheochromocytoma.

## 1. Introduction

Pheochromocytomas are very rare functional catecholamine secreting tumors [1]. Eighty-five percent of pheochromocytomas emerge from adrenal medulla chromaffin cells and the rest of the extra-adrenal paragangliomas originate from sympathetic ganglia [2]. Paragangliomas represent less than 0.2% of hypertensive patients [2]. Although a high index of suspicion is essential, the diagnosis of pheochromocytoma is less likely in the absence of the classic symptoms and laboratory findings.

The incidence rate of pheochromocytoma is 0.8 per 100,000 person–years [3,4]. Usually, a sporadic pheochromocytoma is diagnosed based on presenting symptoms or discovered incidentally on Computed Tomography [CT] scan.

Approximately half of the patients with pheochromocytomas present with the paroxysmal classic triad, which includes episodic headaches, sweating and tachycardia [5,6]. About 50% of the patients have paroxysmal hypertension, the rest either have essential hypertension or normal blood pressure and 10% of pheochromocytomas are metastatic.

In this report, we describe a case of a 70-year-old male diagnosed with a recurrent metastatic pheochromocytoma. When our patient was 60 years old, he was diagnosed with a right adrenal pheochromocytoma, which was treated by laparoscopic adrenalectomy. We report a rare case of metastatic pheochromocytoma recurrence in a patient 10 years later from the age of 60.

## 2. Case

A 70-year-old male with a significant past medical history of adrenal pheochromocytoma status post partial right adrenalectomy ten years ago, atrial fibrillation, hypertension, hyperlipidemia, was brought to the hospital with elevated blood pressure readings and palpitations.

Ten years earlier, at age 60, this patient was brought to the hospital after he was found unresponsive in his apartment. He was admitted to the ICU (Intensive care unit) and was subsequently diagnosed with a stroke. During this hospital stay, he was found to have a right adrenal nodule which was diagnosed with a pheochromocytoma with positive synaptophysin/chromogranin/S100. He underwent a laparoscopic partial right adrenalectomy and was discharged home once his symptoms subsided. He followed up with his primary care provider regularly and his home blood pressure readings remained around 120/77–130/92 mmHg.

Approximately ten years later, he returned to the hospital with palpitations and elevated blood pressure readings with systolic blood pressure of >=200 mmHg. He was diagnosed with another stroke. This time, he developed residual weakness and required the use of a walker due to unsteady gait. He was also diagnosed with atrial fibrillation and was placed on anticoagulants. He was discharged home and regularly saw his primary care physician and attended anticoagulation clinic. He sustained a fall at home one day while walking without a walker and hit his head. There was no loss of consciousness. He went to a clinic four days later where he was noted to be short of breath, pale with extremely high systolic blood pressure of 200 mmHg. Following his admission, he had INR (international normalized ratio) of 4 and hemoglobin of 7 g/dl. He was given 1 unit of red blood cells (RBC) and 2 units of fresh frozen plasma (FFP). Anemia work up showed Fe 55 µg/dL,% saturation 27%, TIBC 207 μg/dL, ferritin 193 ng/mL, B12 546 ng/mL and folate 17 ng/mL indicating anemia of chronic disease. He also had fever and leukocytosis with WBC of 15 µL and was diagnosed with pneumonia and treated with meropenem and doxycycline. His course was further complicated by atrial fibrillation with a rapid ventricular rate. He was put on alpha and beta-blockers; and transferred to subacute rehabilitation under our care for further management. He remained hypertensive at this time which prompted a workup for secondary causes of hypertension.

His past medical history also included heart failure, chronic kidney disease, type 2 diabetes mellitus, anemia, anosmia, hearing loss, vitamin D deficiency, colonic polyps, depression, onychomycosis, hearing impairment, myopia and cataracts.

Before admission, he was independent for all his activities of his daily living. His family helped him with the instrumental activities of daily living. He had 2–3 falls in the past year.

His family history is notable for breast cancer in his sister and liver cancer in another sister. One sister had a kidney and liver transplant. No family history of colorectal cancer, multiple endocrine neoplasia syndrome or other endocrine-related anomalies were reported.

His past medication history includes metformin, escitalopram, apixaban, melatonin, metoprolol and simvastatin. He was started on insulin at the hospital for his blood glucose maintenance.

**Figure 1:**
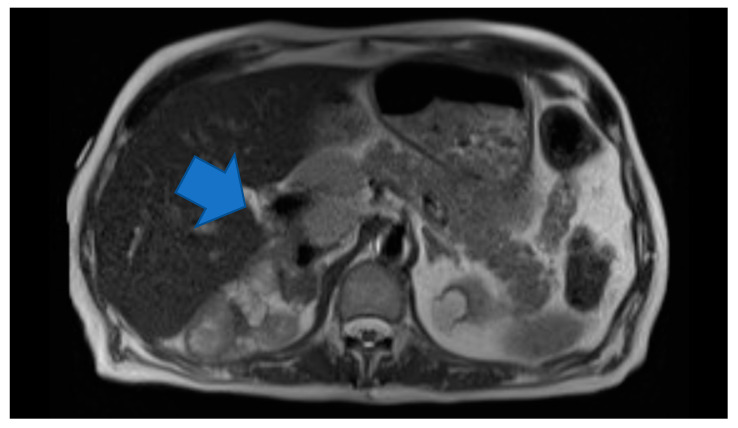
Axial HASTE. Large multilocular heterogeneous mass in the right adrenalectomy bed and large regional lymph nodes causing Inferior vena cava (IVC) compression.

**Figure 2:**
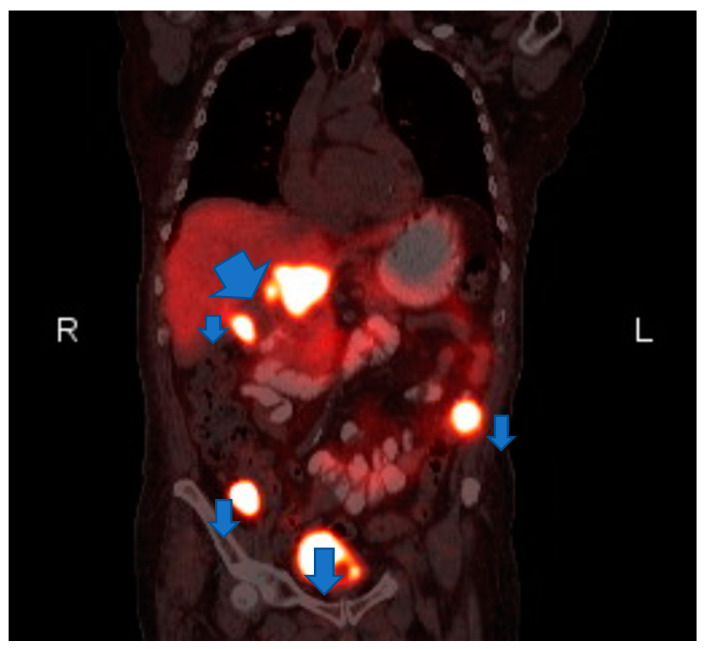
Coronal Postitron Emission Tomography/Computed Tomography (PET/CT). gallium-68 DOTA-DPhe1, Tyr3-octreotate (^68^Ga)Ga-DOTA-TATE Abnormal gallium dotatate accumulation in the right adrenalectomy bed, upper abdominal lymph nodes and two mesenteric nodules.

Laboratory testing revealed anemia (hemoglobin 9.2 g/dl) and hyponatremia (sodium 131). His urine metanephrine was 556 mcg/24 h, normetanephrine was 22,624 mcg/24 h and creatinine was 2.56 mg/dl. His INR was 4.26 which was high. On imaging, there was an abdominal mass on the right upper kidney as well as an abdominal hematoma. Computer tomography (CT) of abdomen showed left lower abdominal wall hematoma and prominent multilobulated soft tissue mass within the right retroperitoneum superior to the right kidney in the region of right adrenal gland involving the liver, vena cava and kidney. Magnetic resonance imaging (MRI) of abdomen and pelvis showed postoperative changes after right adrenalectomy with 7.8 cm heterogenous multilocular enhancing mass in the surgical bed abutting the right posterior wall and close to the right kidney and Inferior vena cava (IVC). The mass and the enlarged regional lymph nodes together caused marked compression of the IVC. Heterogeneity and enhancing focus in the posterior right hepatic lobe indicated the invasion of the right adrenal gland. (Figure 1) Positron emission tomography (PET) gallium-68 DOTA-DPhe1, Tyr3-octreotate ((^68^Ga)Ga-DOTA-TATE) imaging showed liver and mesentery metastases. (Figure 2) He was diagnosed with right metastatic malignant adrenal lesion surrounding IVC along with the syndrome of inappropriate antidiuretic hormone (SIADH).

He was started on the alpha-blockers to control hypertension. After 36 days of admission, he underwent exploratory laparotomy, resection of retroperitoneal masses and an appendectomy. Findings during surgery were omental tumor, tumor at the tip of the appendix, tumor at the right lateral side of the liver; posterior liver/diaphragm mass; and posterior–porta hepatic mass. Pathologic examination of the masses revealed pheochromocytoma and paraganglioma of omental mass, appendix, diaphragm, sub-gall bladder nodule, right portal vein nodule, pericaval and retroperitoneal mass and retro portal mass. Chromogranin and S100 immunostains were positive.

After surgery, the patient was transferred to the intensive care unit for monitoring. Three days after his surgery, the patient developed shortness of breath and bradycardia and had a pulseless electrical activity arrest. A code was run for 13 minutes, the airway was secured, and the patient had the return of spontaneous circulation. After one day, pupils became unequal overnight. CT imaging was negative for brain stem hemorrhage and EEG (electroencephalogram) was unremarkable for any abnormalities. However, ten days after the arrest, patient’s MRI of the head showed diffuse ischemic encephalopathy. He needed continuous ventilatory and circulatory support. The patient showed no improvement in his neurologic status. After 15 days, palliative care was consulted. A goals of care discussion was conducted with family in the wake of poor prognosis and the family opted for hospice style of management. The patient was extubated and provided comfort care and transferred to the hospice unit. He died two weeks later.

## 3. Discussion

Pheochromocytomas are rare tumors that were first described by Fränkel in 1886. Although most tumors are benign and are treated with surgical resection, metastatic transformation can occur decades later [6,7,8]. About 95% of catecholamine producing tumors are located in the abdomen, 85–90% of those are intra-adrenal and 5% to 10% are multiple [9]. Ten to fifteen percent of catecholamine producing tumors are extra-adrenal paragangliomas. About 10% of catecholamine producing tumor have a metastatic potential and for this reason regular follow-up is mandatory for all patients that had resection of a pheochromocytoma [1].

Due to the production of epinephrine, norepinephrine and dopamine, the classic triad of sweating, headache and tachycardia can occur. Sudden spells of paroxysmal hypertension are a self-limiting symptom of pheochromocytoma [10].

Pheochromocytoma, especially recurrent pheochromocytoma, is very rare at an older age. The average age of diagnosis is 47 years old for sporadic tumors not associated with hereditary causes. The average size of tumor 4.9 cm [4]. Incidence is equal in both men and women [10]. In this case report, we present a 70-year-old male with a recurrent pheochromocytoma with metastatic transformation. Based on the literature review, there were few case reports of metastatic pheochromocytoma in the elderly. An 81-year-old male Asian was found to have a giant retroperitoneal tumor, which measured 9.8 × 13.5 × 10.6 cm [11]. Moreover, an 84-year-old male had a large left suprarenal mass 8.1 × 8.7 × 9.4 cm [12]. In contrast to cases reported, our patient underwent laparoscopic right adrenalectomy at an earlier age. After a decade, our patient presented with metastatic pheochromocytoma. The transformation from benign pheochromocytoma to metastatic is a very rare occurrence. Metastatic adrenal pheochromocytoma and extra-adrenal paraganglioma represent 10% to 20% of pheochromocytoma cases [13,14]. The World Health Organization’s definition of metastatic pheochromocytoma requires the presence of metastasis in the non-chromaffin area [15,16].

According to literature, clinical predictors for metastatic transformation of pheochromocytomas are tumor size and location. A tumor size of greater than 5 cm and extra-adrenal tumor location possess greater risk of metastatic transformation. In contrast, our case shows the location of the tumor to be adrenal– in which case, metastatic transformation risks include looking at dopamine secretion, other secretory molecules and tumor cell necrosis [17,18,19,20,21].

The task force by Endocrine Society clinical guidelines subcommittee(CGS) guidelines recommend initial biochemical testing of plasma free or urinary fractionated metanephrines. The positive results are followed up by CT as initial test and MRI for patients with metastatic disease and when radiation exposure needs to be limited. They also recommend genetic testing for all patients. Perioperative blockade with alpha adrenergic receptor blocker and preparation including high sodium diet and fluid intake was recommended. Minimally invasive adrenalectomy was recommended for pheochromocytoma. Lifelong follow up is also recommended for recurrence and metastasis [22].

For our patient, plasma free or urinary fractionated metanephrines were screened. Since he was positive CT follow up was also done. However, 123I-metaiodobenzylguanidine (MIBG) scintigraphy and genetic screening was not done in this patient. He underwent a preoperative alpha-adrenergic receptor blockade to reduce postoperative cardiovascular complications. We also did a 18F-FDG PET/CT scanning on the patient to test for metastasis.

The 123I-MIBG or (131I-MIBG scintigraphy were used in nuclear medicine to detect pheochromocytoma [23]. Currently 123I-MIBG examination is recommended for patients with metastatic paragangliomas detected by other imaging modalities and when radiotherapy using 131I-MIBG is planned [24]. (18F-FDG)-PET shows high sensitivity (near 97%) and specificity (next to 90%) in detecting metastatic pheochromocytoma, whereas 18F-DOPA-PET (18-F fluoro-dihydroxyphenylalanine positive emission tomography) is less accurate in diagnosing metastatic pheochromocytoma (<=45%) [24]. We ordered whole-body (68Ga)-DOTA-TATE neuroendocrine PET/CT scan to detect metastases in our patient.

Germline mutations contributes to the tumor growth and progression. Around 40% of the pheochromocytomas and paragliomas (PPGL) carry germline mutation [25]. It has the highest degree of heritability of all the tumors [26]. Based on the gene expression signature, they are classified into two clusters. Cluster 1 genes that are associated with hypoxic response (von Hippel–Lindau (*VHL),* succinate dehydrogenase A *(SDHA),* succinate dehydrogenase B *(SDHB),* succinate dehydrogenase C *(SDHC)*, succinate dehydrogenase D *(SDHD) and* succinate dehydrogenase *AF2 (SDHAF2) and* hypoxia-inducible transcription factors *(2A HIF2A)*) while cluster 2 tumors (rearranged during transfection (*RET),* neurofibromatosis 1 *(NF1),* transmembrane protein 127 *(TMEM127) and MYC-associated factor X (MAX))* mutations that activates kinase signaling and protein translation. PPGLs show high degree of heterogeneity. Genetic screening algorithms using conventional Sanger sequencing (SS) as well as targeted gene panels (TGPs) were used as a diagnostic tool for detecting PPGL. Conventional Sanger sequencing (SS) are useful in syndromic presentation. Next-generation sequencing (NGS) technology is another valuable tool for genetic screening, but the test is expensive and due to the ethical concerns with incidental findings, they are mostly used in research settings. Targeted gene panels (TGPs) screens for genes that are systematically excluded in SS studies. DNA samples for these studies may the obtained from blood, saliva, DNA samples formalin-fixed paraffin-embedded (FFPE) tissue and frozen tumors [25,27].

The treatment approach to patients with pheochromocytoma must be individualized and this is especially true for the elderly. Alpha- and beta-adrenergic blockers and calcium channel blockers can be used to control the hormone-related symptoms. Current perioperative management of pheochromocytoma includes preoperative antihypertensive therapy, including alpha blockade for the negation of alpha 1 mediated vasoconstriction and beta1 mediated tachycardia and inotropy [22]. The alpha blockade is started 10–14 days before surgery to control blood pressure and expand the highly contracted intravascular volume. Successful alpha blockade is reflected by normalizing blood pressure and mild orthostasis. Beta receptor antagonists are later added to counteract tachycardia due to non-selective alpha blockade and vasodilation induced tachycardia [22] Control of the catecholamine excess signs and symptoms by medical therapy has proven to decrease the intra and postoperative cardiovascular complications. However, our patient experienced cardiovascular complication post-surgery despite being on alpha blockade with doxazosin before surgery.

All patients who undergo surgery must have a thorough cardiac evaluation. Patients who have longstanding tumors can develop severe dilated cardiomyopathy with varying degrees of heart failure, increasing perioperative risk. Our patient with atrial fibrillation, had and electrocardiogram (EKG) and echocardiogram (ECHO) before surgery, but still developed pulseless electrical activity (PEA) arrest two days post-surgery, further emphasizing the need for thorough cardiac evaluation and follow up.

Pheochromocytomas are often radio and chemo resistant. However, the presence of somatostatin receptors in chromatin cells allow for the use of somatostatin analogs for localization during diagnosis and also the treatment of tumor. In patients who express somatostatin receptors and also have a positive 123I-MIBG scan, palliative debulking therapy with 131I-MIBG therapy can be used [28]. The 131I-MIBG therapy adverse events include leukopenia, thrombocytopenia and bone marrow toxicity. In our patient, the perioperative blockade was provided along with scheduled debulking surgery.

The risk-benefit analysis of surgical versus non-surgical management must be thoroughly assessed on a case-by-case basis. Non-surgical modalities can be used in conjunction or as the primary mode of treatment in some cases. Directed trans arterial chemoembolization, percutaneous tumor ablation and external beam radiation are some of the available non-surgical modalities. In surgery, R0 or R1 resection is a better approach compared to R2 or surgical debulking. A multidisciplinary approach is the best way for the management of this complex tumor. In our patient, due to the extensive spread of the tumor, surgical debulking was done.

The overall 5-year-survival rate of patients with metastatic pheochromocytoma ranges between 34% and 60% [29].

## 4. Conclusions

In conclusion, pheochromocytoma is an extremely rare tumor and the occurrence and reoccurrence in the elderly is even rarer. A low threshold for the tumor suspicion must be maintained if there is hypertension or cyclic hypotension and hypertension. Surgery is the standard treatment of the tumor with medication for symptom control.
